# Selections

**Published:** 1896-05

**Authors:** 


### Selections.

### PARACHLOROPHENOL.

Parachlorophenol is a new antiseptic introduced by Nencki, and described therapeutically by Professor Girard, of Bern, in *Revue Médicale de la Suisse Romane* (July 20, 1895). The product occurs in crystalline form, is readily soluble in alcohol or ether, but nearly insoluble in water (only up to one and three-tenths per cent.). Its bactericidal effect on anthrax spores has been verified by Karpow, of Dorpat; its two-per-cent. solution is only slightly weaker than a one to one thousand sublimate solution, but considerably stronger than a five-per-cent. phenol or cresol solution.

Girard records his use of parachlorophenol in nearly two hundred cases. The one-per-cent. solution had a favorable effect on fresh wounds (two per cent. seemed much more irritating), and in no case did irrigation, even of large cavities (goitre, etc.), lead to toxic symptoms; it sterilizes instruments without affecting them; and it is less irritating to the skin than either sublimate or phenol. Girard states that he prefers parachlorophenol to solveol and lysol because of its more exact chemical composition.—*Notes on New Remedies.*

### DISINFECTANTS.

The diluting of disinfectants with alcohol, glycerin, and oil makes them ineffectual. Dr. Lenti, of the Hygienic Institute of Naples, has found that corrosive sublimate dissolved in alcohol has proved useless; even in one to two hundred and fifty solution on spores which were placed in solution for forty-eight hours, their virulence was only weakened. By adding ten per cent. water to the alcohol the germs were destroyed in a one to one thousand solution. A two-per-cent. solution of corrosive sublimate in pure glycerin was useless even after subjecting the spore to it for four days. By adding forty per cent. water they were destroyed in a solution of two to one thousand in twenty-four hours. A ten-per-cent. solution of carbolic acid in alcohol is useless, and remains so even up to fifty per cent. By adding eighty per cent. water the germs were destroyed in forty-eight hours. A ten-per-cent. solution of carbolic acid in glycerin proved ineffectual even after seventy-two hours; ten per cent. water added did not change it; but after eighty per cent. water was added it destroyed the germs in forty-eight hours. A twenty-per-cent. solution of carbolic acid in oil, and a ten-per-cent. solution of lysol in oil, are both useless.—*Zahntechniche Reform.*

---

### A NEW HÆMOSTATIC.

In the *Lyon Médical* for February 17 there is an abstract of an article which appeared in the *Therapeutische Wochenschrift* for January 6, in which the author, Dr. Hederich, of Heidelberg, recommends a new hæmostatic called ferripyrine. This substance is a definite combination of iron perchloride and antipyrine, under the form of a reddish orange-colored powder which is easily soluble in cold water. It has several appreciable advantages over iron perchloride, and has neither its caustic qualities nor its bad taste; its hæmostatic properties are, moreover, superior to those of the perchloride. In a case of profuse hemorrhage caused by a very vascular myxoma of the nasal cavity, the application of two tampons saturated with a twenty-per-cent. solution of ferripyrine was sufficient to arrest the flow of blood completely. It may also be employed internally in doses of eight grains. Dr. Hederich thinks that it should be tried in cases of blennorrhagia in injections of one-per-cent. or one-and-one-half-per-cent. solution.—*New York Medical Journal.*

